# Perceived family support among older persons in diabetes mellitus self-management

**DOI:** 10.1186/s12877-018-0981-2

**Published:** 2018-12-19

**Authors:** Niko Dima Kristianingrum, Wiwin Wiarsih, Astuti Yuni Nursasi

**Affiliations:** 1 School of Nursing, Faculty of Medicine, Universitas Brawijaya, Malang, Indonesia; 2 Faculty of Nursing, Universitas Indonesia, Depok, Indonesia

**Keywords:** Family support, Self-management, Diabetes mellitus, Older persons

## Abstract

**Background:**

The aging process has functional consequences for older persons, such as degenerative processes of the pancreas resulting in diabetes mellitus. The increasing age of the population will eventually lead to increasing health problems of older persons, including diabetes mellitus. Diabetes mellitus is a chronic disease that requires long-term care through self-management. Diabetes self-management in older persons is influenced by family support as the main support system. This study aimed to explore perceived family support by older persons in diabetes mellitus self-management.

**Methods:**

This study applied descriptive phenomenology method. The data were collected through in-depth interviews. There were nine older persons with diabetes mellitus as participants. Data consisted of in-depth interview recordings and field notes. Data were transcribed and analyzed using Colaizzi’s method.

**Results:**

The results identified that family support as perceived by older persons included daily activity assistance, assistance with obtaining health services, food preparation, financial support, attention, guidance, and problem solving. The response to family support was pleasure as expressed by the older persons.

**Conclusions:**

Physical and economic limitations were a significant hindrance to self-management of diabetes mellitus in older persons; therefore, they require family support to optimize their independence. The results of this study highlight the importance of family support in diabetes mellitus self-management in older persons.

## Background

The increasing life expectancy has impacted on the proportions of older persons. Globally maintaining an older person’s health and quality of life is a challenge with the increasing numbers [1]. Older persons experience an aging process that impacts on their physical, social, psychological, and spiritual well-being [2]. They also face degenerative processes in their body systems, including a decrease in pancreas function [3]. Data for an increasing diabetes prevalence showed 171 million cases in 2000, 382 million in 2013, and an estimated 592 million in 2035 [4].

Diabetes can affect patients, their family, and their country. Patients could have a reduction in their physical function, negative emotional responses, and decreased social interaction and productivity. It also causes changes in economic functions and the family role. Diabetes also requires a great deal of health expenditure for long-term care. In the USA, the cost for diabetes care reached 174 billion US dollars in 2007 and increased to 245 billion US dollars in 2012 [5].

Since diabetes mellitus (DM) is a chronic condition it requires long-term self-management. Self-management is the basis of treatment for DM and includes controlling blood sugar levels to prevent or reduce the severity and complications. DM self-management is influenced by enabling and inhibiting factors. Enabling factors consist of family support, relations with a health professional, and social interaction, while inhibiting factors are drug reluctance, food culture, and assurance limitation [6].

The family is main social support for patients [7]. Family support can be divided into four types: instrumental, information, reward, and emotional [8]. DM self-management should be based on a nursing model by integrated knowledge and experiences of the patient. The nurse should carefully observe the perspective of the patient to help solve DM self-management problems [9]. DM self-management is independent and based on the individual. It is also influenced by family support. Therefore, it is important to assess and explore the perception of family support by the older person to succeed in DM self-management. The aim of this study was to explore the meaning of the perceived family support by older persons in DM self-management.

## Methods

This study used a qualitative research by descriptive phenomenology approach. The sampling procedure in this study used purposive sampling techniques with a criterion sampling category. Purposive sampling is the technique of sampling according to criteria that have been set in accordance with the intent and purpose of research [10, 11]. Criterion sampling selects participants who meet the criteria that have been established in accordance with the phenomenon studied, as well as a possible approach to identify and understand participants who are rich in information about the experience of a phenomenon [12]. The participant’s inclusion criteria were ≥ 60 years old, have acquired information (undergoing DM treatment at home, ever received information about DM care), the ability to tell their experience (can speak Indonesian language, not having impairment in speech, hearing, and cognitive), have direct experience, are willing to be interviewed, and are willing to participate [11].

The researcher considered three ethical principles according to the Belmont Report: beneficence, respect for human dignity, and justice [12]. The principle of beneficence emphasized that researchers should minimize hazards and maximize the benefits of research for participants [12]. The principle of respect for human dignity gives the participant the right to decide the choice (self-determination) and the right to get a full explanation (full disclosure) [12]. The researcher explained the purpose, benefits, procedures, and role of the participant, and then gave the participants the opportunity to determine whether or not to volunteer to participate in the study. The researcher then grants eligible participants the right to participate or not. The researcher gave the participants the freedom to resign from the research. The researcher also ensured the absence of any sanctions against participants who are not willing or those who resigned. The researcher obtained written informed consent from the participants. The researcher also contracted the time and place for the interview with the participants prior to the interview so that the participants were not put out by the researcher. The principle of justice was to give equal treatment without distinction for ethnicity, religion, or class, and does not distinguish socioeconomic and educational status, including not offending the basic weaknesses of participants [12]. The researcher also needs to pay attention to the principle of confidentiality for data or information submitted by participants and which will only be used for research purposes [11].

Data collection, data processing, and data analysis were conducted in May and June 2015. Researchers employed researchers themselves as the primary data collection tool. Other data collection tools used in this research were interview guidance, field notes, and a digital audio recorder. Researchers conducted semistructured in-depth interviews with prior guidance and interview assistance. Prior to the interview, they were tested on two older persons with diabetes who were not participants. This was to train the researchers to respond and to dig deeper into the respondent’s perspective. The researcher encouraged participants to talk about DM self-management experiences by asking open-ended questions in a one-to-one interview at nine participant homes with a face-to-face approach. No subject refused to participate. Participants were four men and five women. They were 60–80 years old. Two participants had completed elementary school, two participants had completed junior high school, three participants had completed senior high school, and one had completed a bachelor’s degree. Two participants were widows, and seven participants were married. Five participants were Javanese, three participants were Betawinese, and one was Balinese. Two participants lived with a with spouse, one participant lived with children, and four participants lived with their spouse and children. Participants were diagnosed with diabetes for between 6 months and 20 years. In-depth interviews were conducted for 50–90 min with each participant. Field notes were made during interview. The data were analyzed by Colaizzi’s method since this provides deeper access to the implicit or explicit meanings of participant descriptions. Colaizzi also involves clarification to the participants regarding the results of the analysis [10, 12]. The researcher reworded and clarified the statements for further investigation of topics introduced by the respondent. Transcripts were returned to the participants for comment. The researcher read the whole transcript, examined each description and separate significant statements, formulated the meaning of each significant statement, organized or categorized each unit of the meaning into the theme, and formulated or integrated each theme into a full description as a clear statement. Data saturation was discussed by the researcher with two supervisors. Supervisors acted as external analyzers and analyzed all the data to produce an inquiry audit. The last stage was a final validation by showing the results of the analysis to the participants to give feedback on the findings.

## Results

**Fig. 1:**
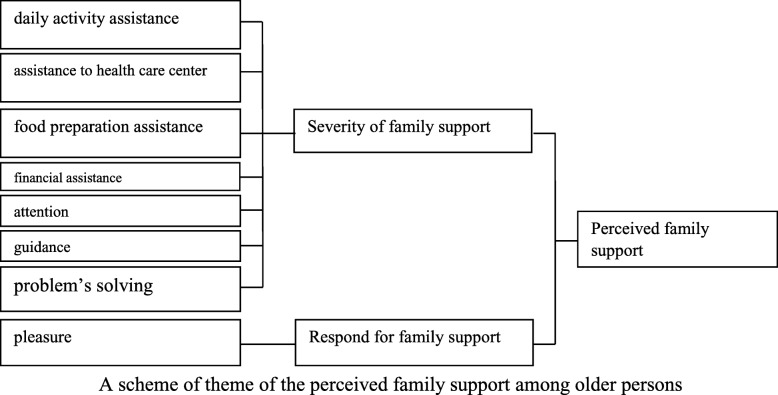
A schema of the perceived family support among older persons. Perceived family support was shaped by two themes: various areas of family support and response to family support. The areas of family support consist of daily activity assistance, assistance with the health center, food preparation assistance, financial assistance, attention, guidance, and problem solving. The response to family support was pleasure

We identified several family support areas perceived by older persons along with their response to such support areas. A thematic scheme of perceived family support among older persons is described in Fig. 1. The family support included daily activity assistance, assistance with health services, food preparation, financial support, attention, guidance, and problem solving.

### The levels of family support

#### Daily activities assistance

Participants received help from the family to perform daily activities. Participants expressed examples such as: “I cannot do ironing and washing, all were done by my daughter and cooking are mostly done by my husband. My family help me when I want to take a bath” (P1) and “My wife prepares all my needs, if I want to take a bath or to eat, all were done by my wife” (P9).

#### Assistance with health services

Participants received family support to access healthcare services. Participants expressed the following statements: “If I want to check my health, my son accompanies me to the clinic. If I was ill, I would be taken to hospital or public health centers immediately” (P2) and “My son accompany me to the clinic, and sometimes by my husband, it doesn’t matter” (P1).

#### Food preparation

Participants got help from the family for food preparation. Their family cooked and prepared meals for the participants. This was disclosed through the following statements: “So all the food cooked and prepared by my wife, I just eat when foods already prepared” (P6) and “My daughter cooked for me” (P2).

#### Financial support

Participants received financial support from the family and they also have their own arrangements. Participants said that their family helped them to buy medicine and daily necessities. Participants expressed example phrases as follows: “All my financial needs were already covered by my son” (P2) and “He bought medicine using his money, all were paid by my son” (P7).

#### Attention

Participants received psychological support from the family. Participants said that their family became more attentive and often reminded them to consume food and take their medicine. Some phrases that were expressed by the participants are as follows: “My young daughter worried about me...if I’m sick, she gave more attention about my food” (P2) and “She gave great attention to me. She doesn’t want me to be tired. She took care of me. She liked to remind me for taking medicine” (P7).

#### Guidance

Two participants received advice from their family in performing self-management. P7 said that her son prohibits her from doing house chores and reminds her not to get tired. P5 said that her sister advised her to check her blood glucose level. The participant statement was expressed as follows: “I wasn’t permitted to do house chores... I am not tired” (P7) and “My sister asked me to try to go to hospital for blood sugar checking” (P5).

#### Problem solving

Two participants obtained solutions from their family to face DM self-management. P6 stated that, when his blood sugar rises, his wife suggests that he should not to think about it because it is the usual thing in people with DM. Meanwhile, P3 said that his wife provided a solution by often reminding him to maintain his diet as necessary. Participants expressed the following statements: “My wife told me to not be stress out, she said it’s usual if blood glucose was high or normal. It is more important to control my diet, taking medicine and minimizing rice consumption. It’s important to eat variety of vegetables, and it is better to exercise” (P6) and “I often get reminded about my diet, my wife tends to be afraid I will be sick” (P3).

### The response to family support

Participants responded positively about their family support. Older persons were happy and grateful to receive the family support. This is reflected on participant statements regarding these matters as follows: “I am grateful anyway for having children and husband who gave me attention” (P4), “I’m happy that my wife take care of me” (P3), and “I am really happy to get attention but unfortunately it cannot be everyday” (P5).

## Discussion

This study describes the various forms of family support that are provided by the family and the response of the older persons to this support was good. Family support as perceived by older persons involved daily activities assistance, assistance with health services, food preparation, financial support, attention, guidance, and problem solving. Daily activity assistance from the family included preparation for bathing, dressing, washing, ironing, and assistance with social activity. Assistance with the health service including transportation to the healthcare center for a health status check and blood glucose level check. Older persons also attained financial support from the family, including money for medicine and other daily necessities of life. The also received family’s attention for such things as concerns and advice from the family, reminders to take medication, diet maintenance, and regular blood sugar checks. The older persons also accepted solutions to their problems. The responses of the elderly to the family support were that they were very pleased and grateful that they were able to therefore perform self-management well.

A previous study by Ismonah supports our results that there is a relationship between family support and self-management, where patients who received family support were likely to be 10 times better at performing self-management [13]. Other support was emotional and financial. These were divided into four types: instrumental, information, awards, and emotional [8]. Siriwatanamethanon and Buatee in 2013 [14] reported that older persons with DM can no longer work and remain nonproductive at home. Therefore, older persons with DM need family support. However, the older persons were still physically active, with two still work for a living and some were still able to perform daily activities independently [14].

Older persons with DM are considered to be a vulnerable population that has experienced declining physical function that impacts on their role and activity. These limitations restrain them from optimally performing activities to meet financial needs. Therefore, older persons with DM require family support such as economic support, information support, instrumental support, and psychological support.

Moser et al. in 2008 [15] explained that DM self-management should be a part of family life. All participants in their study stated that their families supported them to manage themselves in various ways [15]. Bhattacharya in 2012 [16] in his research explained that most of the participants stated that their family supported them to perform self-management by helping prepare meals, taking medication, and advice to exercise. However, most participants stated that the family was not the main source of support as they lived alone or family members did not take care of them. Women attempted to perform self-care, including diet, exercise, and medication. On the other hand, men felt depressed because of loneliness and their families treated them as having a lifelong illness [16]. This finding highlights the cultural difference between our study and that of Bhattacharya.

In this study, the family is the main support for older persons to help them perform self-management. Most of older persons felt the support of the family, especially those who lived with their family including a spouse, son, daughter, and grandchildren. They also were happy if they were observed by family members. They also hope their proximity to the family could be maintained, even those who lived far away. This is supported by Badriah [17] explaining that, in Indonesia, caring for older persons with DM is a form of obligation, worship, and reciprocation, as well as the responsibility of children to their parents. It also means that children adhere to the teachings of religion and culture [17].

## Conclusions

Older persons have some DM self-management limitation; therefore, they require family support to optimize their independence. The experience of self-management of DM among older persons provides a wide range of useful information about family support among the elderly population. There are different types of family support and different responses to family support.

## Abbreviations

DM: Diabetes mellitus
